# A Prolonged Presentation of May-Thurner Syndrome With Extensive Deep Vein Thrombosis

**DOI:** 10.7759/cureus.84295

**Published:** 2025-05-17

**Authors:** Joseph Marcuccilli, Nathan Jatczak, Bradley Trotter

**Affiliations:** 1 Medicine and Surgery, A.T. Still University, Kirksville, USA; 2 Medicine, Fort Wayne Medical Education Program, Fort Wayne, USA

**Keywords:** atypical back pain, deep vein thrombosis (dvt), deep venous stenting, leg swelling, may-thurner syndrome

## Abstract

An 18‐year‐old female presented to the emergency department with progressive bilateral lower extremity pain and swelling. She reported a two‐week history of back pain radiating down both legs, initially treated with steroids and muscle relaxants without improvement. Initial imaging, including an MRI and hip X‐rays, was unremarkable. She subsequently developed worsening left leg swelling, prompting further evaluation for deep vein thrombosis (DVT). She had no history of oral contraceptive use, recent travel, surgery, or prolonged immobility. A family history of “thick blood” in her mother and aunt raised concerns for an underlying hypercoagulable state. Physical examination revealed diffuse bilateral leg swelling with warmth and erythema. A venous duplex ultrasound confirmed extensive left lower extremity DVT, which was further characterized on CT as extending from the common femoral vein through the iliac veins and infrarenal inferior vena cava. Imaging also identified compression of the left common iliac vein by the right common iliac artery, consistent with May-Thurner Syndrome (MTS). Management included systemic anticoagulation, catheter-directed thrombolysis, venous stenting, and Inferior Vena Cava (IVC) filter placement. This case highlights the importance of recognizing MTS as a potential cause of unprovoked DVT in young patients, emphasizing the need for early diagnosis and intervention to prevent long-term complications.

## Introduction

An 18‑year‑old female presented to the emergency department with progressive bilateral lower extremity pain and swelling. She reported a two‑week history of back pain radiating down both legs, initially treated with steroids and muscle relaxants without improvement. Initial imaging, including MRI of the lumbosacral spine and hip X‑rays, was unremarkable. Two days after the presentation, she developed worsening left leg swelling, prompting evaluation for deep vein thrombosis (DVT). She had no history of oral contraceptive use, recent travel, surgery, or prolonged immobility. A family history of “thick blood” in her mother and aunt raised early concern for an inherited hypercoagulable state; subsequent thrombophilia testing (including factor V Leiden, prothrombin gene mutation, and antiphospholipid antibodies) returned negative and did not alter acute management, but remains under longitudinal surveillance given her family history. Physical examination revealed diffuse bilateral leg swelling with warmth and erythema. Venous duplex ultrasound confirmed extensive left lower extremity DVT, which contrast CT further characterized as extending from the common femoral vein through the iliac veins and infrarenal inferior vena cava. Imaging also identified compression of the left common iliac vein by the right common iliac artery, consistent with May-Thurner Syndrome (MTS). Management was initiated with systemic anticoagulation (low‑molecular‑weight heparin), followed by catheter‑directed thrombolysis to rapidly restore patency. After successful thrombolysis, a self‑expanding venous stent was placed across the stenotic left common iliac vein to correct the underlying mechanical compression. Given the extensive clot burden and risk of embolization during intervention, a temporary inferior vena cava (IVC) filter was inserted; in this young patient, the filter was planned for removal once clot resolution was confirmed to minimize long‑term device‑related risks. This case highlights the importance of recognizing MTS as a potential cause of unprovoked DVT in young patients, emphasizing early diagnosis and a stepwise interventional approach. Close follow‑up is essential to monitor for key long‑term complications, specifically post‑thrombotic syndrome and chronic venous insufficiency, and to ensure timely removal of temporary devices.

## Case presentation

An 18‐year‐old female presented to the emergency room with complaints of lower extremity pain and swelling. She reported a two‐week history of pain originating in her back and radiating down both legs. Despite receiving steroids and muscle relaxers at a walk-in clinic, her symptoms persisted. MRI and hip X-rays performed at a hospital were within normal limits, and she was discharged without a definitive diagnosis. However, her symptoms worsened with increased swelling in her left leg. A pediatric physician evaluated her and referred her to the emergency department to rule out DVT.

She denied any use of oral contraceptives, recent travel, surgery, prolonged immobility, or prior thrombotic events. She also had no history of diabetes, malignancy, or intravenous drug use. Notably, her mother and aunt had a family history of “thick blood,” although her mother had never required anticoagulation therapy.

Physical exam

On examination, the patient had diffuse bilateral lower extremity swelling, warmth, and erythema, with left-sided calf tenderness on palpation. She reported severe left leg pain with associated pitting edema, making ambulation too painful. The right leg also exhibited moderate pitting edema but was less symptomatic. The patient appeared visibly uncomfortable and in severe pain. A review of systems was significant for the absence of chest pain, shortness of breath, hemoptysis, or palpitations, ruling out signs of pulmonary embolism. She denied recent trauma, prolonged immobility, recent travel, or prior venous thromboembolism. There were no symptoms of systemic infection, such as fever, chills, or night sweats. The patient had no history of easy bruising, abnormal bleeding, or known coagulopathies, though a family history of “thick blood” was noted. She denied oral contraceptive use, recent surgery, malignancy, or intravenous drug use. There were no neurological deficits, including numbness, tingling, or weakness.

Laboratory reports at the time of admission

On admission, the patient’s vital signs were stable, with a blood pressure of 115/68 mmHg, heart rate of 89 beats per minute (bpm), respiratory rate of 15 breaths per minute, temperature of 98.5°F, and oxygen saturation of 100% on room air. Physical examination revealed diffuse swelling in both lower extremities with associated warmth and erythema. A positive Homan’s sign was noted on the left leg, while the patient maintained a normal range of motion with intact distal sensation and pulses. Laboratory investigations showed a white blood cell count of 11.64 ×10³/mcL, red blood cell count of 5.60 ×10³/mcL, hemoglobin of 10.5 g/dL, and platelet count of 236 ×10³/mcL. Coagulation studies demonstrated an activated partial thromboplastin time (aPTT) of 31 seconds, prothrombin time (PT) of 13.4 seconds, international normalized ratio (INR) of 1.19, and fibrinogen level of 71 mg/dL. Antithrombin III activity was reduced at 45, and Anti-Xa unfractionated levels measured 0.18 IU/mL. A pregnancy test (Beta HCG) was negative. Liver and kidney function tests were within normal limits.

Imaging

**Figure 1:**
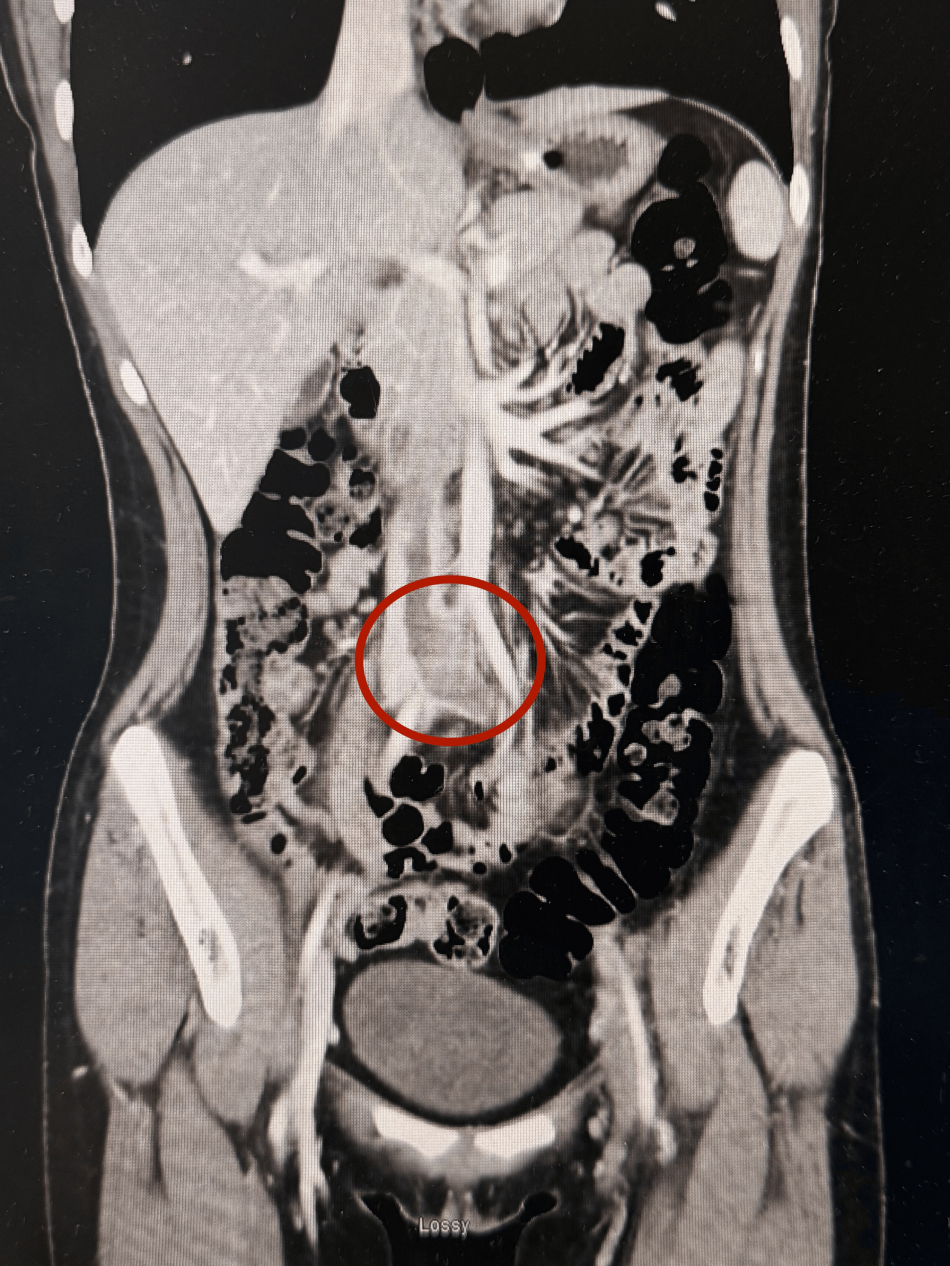
CT angiogram of the abdomen and pelvis (coronal view) Coronal CT angiogram of the abdomen and pelvis demonstrating compression of the left common iliac vein by the overlying right common iliac artery. The red circle highlights the site of thrombosis consistent with May-Thurner syndrome.

**Figure 2:**
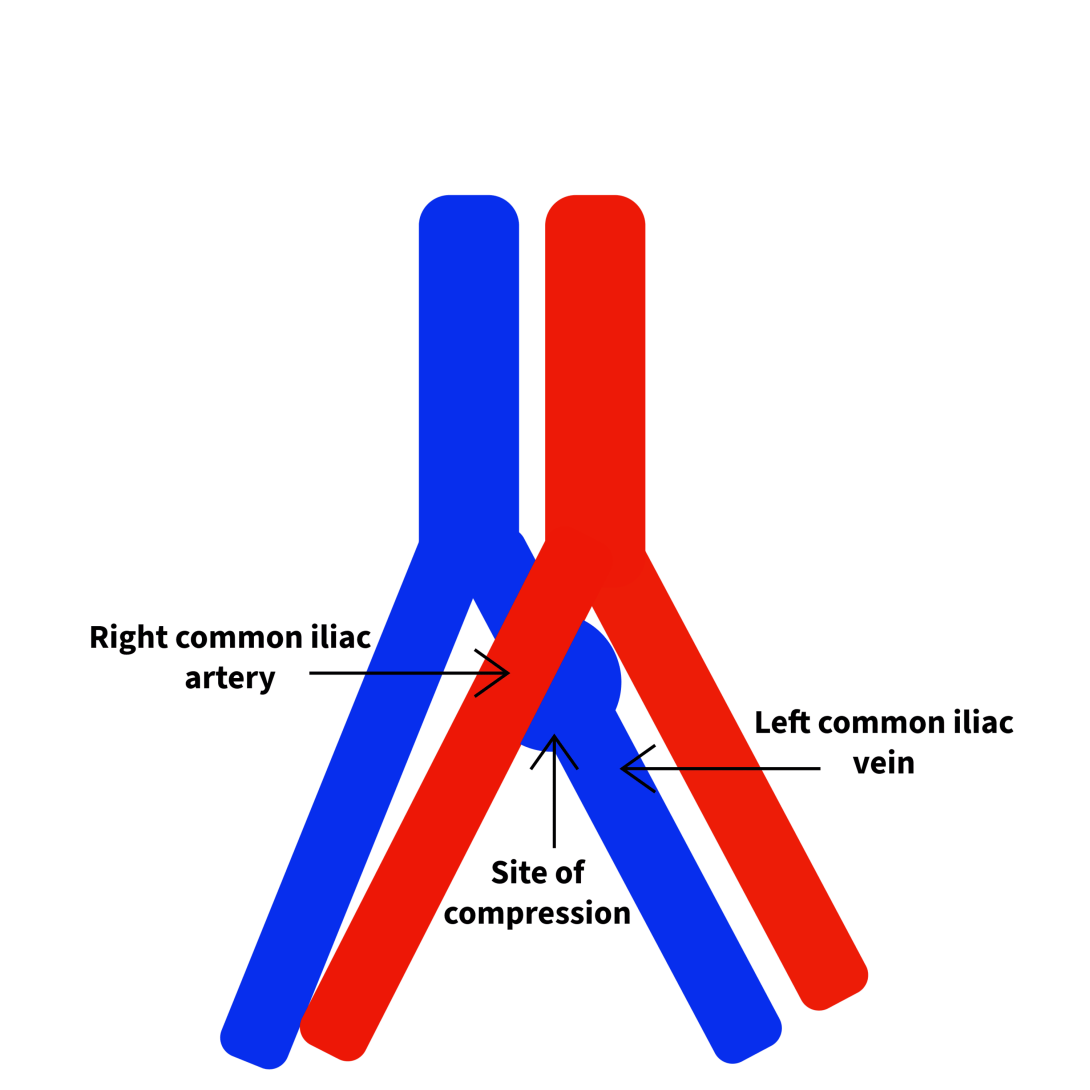
Anatomic variation of May-Thurner Syndrome showing the site of compression of the left common iliac vein The image created by the authors.

Imaging studies confirmed extensive thrombotic involvement of the left lower extremity. Bilateral lower extremity venous duplex ultrasound revealed an extensive deep vein thrombosis (DVT) in the left leg, demonstrating noncompressibility, lack of patency, and absence of spontaneous Doppler flow from the common femoral vein to the left calf veins. A CT scan of the abdomen showed involvement of the left femoral vein, left external iliac vein, left common iliac vein, and infrarenal segments of the inferior vena cava (IVC). Additionally, the right common iliac artery was seen crossing over and compressing the left common iliac vein, a characteristic finding of May-Thurner Syndrome [1]. An electrocardiogram (ECG) showed normal sinus rhythm with sinus arrhythmia, while a chest X-ray (CXR) demonstrated no evidence of an acute cardiopulmonary process. Limited ultrasound of the IVC showed no evidence of venous thrombosis. Based on these findings, the patient was diagnosed with May-Thurner Syndrome with extensive unilateral left lower extremity DVTs. Figure 1 demonstrates the CT angiogram (CTA) of the patient, showing the compression site of the left common iliac vein from the overriding right common iliac artery, and the clot formed secondary to the compression. The image in Figure 2 shows a representation of the CTA.

Procedures

The patient was promptly initiated on anticoagulation therapy with intravenous heparin. Given the extensive clot burden, an IVC filter was placed by interventional radiology (IR) to prevent embolization. Venography of the left lower extremity, left pelvis, and IVC was performed, followed by pharmacomechanical thrombectomy of the left popliteal vein, femoral vein, common femoral vein, external iliac vein, common iliac vein, and IVC. Catheter-directed thrombolysis using the EKOS^TM^ system (Boston Scientific, Marlborough, Massachusetts) was initiated, with catheters placed via the right internal jugular vein and left posterior tibial vein. Subsequent procedures included retrieval of the IVC filter, venoplasty with angioplasty and stent placement in the left common and external iliac veins, and additional pharmacomechanical thrombectomy of the left femoral vein. Pain management was provided with a combination of intravenous morphine, oral acetaminophen, and intravenous ketorolac as needed.

## Discussion

Pathogenesis

The pathogenesis of MTS involves mechanical compression and subsequent endothelial injury, leading to venous stasis and thrombosis. Chronic pulsatile compression results in intimal hyperplasia and fibrosis of the left iliac vein, increasing the risk of thrombogenesis. This aligns with Virchow’s triad, which describes the three primary factors contributing to DVT: venous stasis, endothelial injury, and hypercoagulability [2]. Although many individuals with iliac vein compression remain asymptomatic, those with additional prothrombotic risk factors, such as genetic thrombophilias or chronic inflammatory states, are at greater risk of developing clinically significant DVT [3].

Diagnosis

DVT is typically established using duplex ultrasound, which evaluates venous compressibility and flow. However, ultrasound may be insufficient for detecting iliac vein compression. Computed tomography angiography (CTA) and magnetic resonance imaging with contrast (MRI) are widely used for diagnosing MTS, as they provide detailed visualization of venous compression and collateral circulation [4]. Intravascular ultrasound (IVUS) has recently emerged as a superior tool for identifying venous stenosis and guiding endovascular treatment [5].

Treatment

The primary goal of MTS management is to prevent thrombus propagation, alleviate symptoms, and reduce the risk of recurrence. Initial treatment includes anticoagulation, typically with heparin or low-molecular-weight heparin, followed by long-term therapy with direct oral anticoagulants or warfarin [6]. In cases of extensive thrombosis, catheter-directed thrombolysis (CDT) is often employed to rapidly restore venous patency and reduce the risk of post-thrombotic syndrome [4]. Endovascular intervention is the definitive treatment for MTS, with angioplasty and stenting of the left iliac vein being the standard of care. Stent placement alleviates venous obstruction, preventing recurrent thrombosis and improving long-term outcomes. Studies have demonstrated high success rates and durable patency with this approach [3]. Surgical options, such as venous bypass or iliac artery transposition, are reserved for refractory cases or when endovascular treatment is not feasible.

## Conclusions

May-Thurner Syndrome is an often‑overlooked cause of deep vein thrombosis (DVT), more commonly reported in young female patients, being at least twice as frequent in women as in men, but it can occur across all demographics and should not be excluded solely based on gender. Its nonspecific presentation means many cases remain undiagnosed until significant thrombotic events occur, as up to 22 % of individuals harboring iliac vein compression may be asymptomatic until DVT develops. This case underscores the practical takeaway that MTS should be considered in the differential diagnosis of unilateral lower extremity DVT in young patients without clear risk factors, even when bilateral leg discomfort is reported. Early diagnostic imaging, beginning with duplex ultrasound and proceeding to CT/MR venography and adjunctive intravascular ultrasound (IVUS) for real‑time, high‑sensitivity visualization, enables accurate identification of venous compression and guides intervention. Advances in endovascular therapies, including catheter‑directed thrombolysis and stenting, have greatly improved outcomes, but prompt recognition and intervention are critical to prevent key long‑term complications such as post‑thrombotic syndrome and recurrent DVT. A specific insight from this case, the presentation of bilateral symptoms despite unilateral MTS and the finding of low antithrombin III activity, highlights the importance of objective asymmetry measurements and comprehensive thrombophilia workup in guiding diagnosis and long‑term management.
